# Stem cell Transplantation for Eradication of Minimal PAncreatic Cancer persisting after surgical Excision (STEM PACE Trial, ISRCTN47877138): study protocol for a phase II study

**DOI:** 10.1186/1471-2407-14-168

**Published:** 2014-03-10

**Authors:** Friedrich H Schmitz-Winnenthal, Thomas Schmidt, Monika Lehmann, Philipp Beckhove, Meinhard Kieser, Anthony D Ho, Peter Dreger, Markus W Büchler

**Affiliations:** 1 Department of General, Visceral and Transplantation Surgery, University of Heidelberg, Im Neuenheimer Feld 110, 69120 Heidelberg, Germany; 2 Coordination Centre for Clinical Trials, Building 4410, Voßstraße 2, 69115 Heidelberg, Germany; 3 German Cancer Research Center (DKFZ), Im Neuenheimer Feld 280, 69120 Heidelberg, Germany; 4 Institute of Medical Biometry and Informatics, University of Heidelberg, Im Neuenheimer Feld 305, 69120 Heidelberg, Germany; 5 Department of Medicine V, University of Heidelberg, Im Neuenheimer Feld 410, 69120 Heidelberg, Germany

**Keywords:** Pancreatic cancer, Allogeneic hematopoietic stem cell transplantation, Minimal residual disease

## Abstract

**Background:**

Pancreatic cancer is the third most common cancer related cause of death. Even in the 15% of patients who are eligible for surgical resection the outlook is dismal with less than 10% of patients surviving after 5 years. Allogeneic hematopoietic (allo-HSCT) stem cell transplantation is an established treatment capable of to providing cure in a variety of hematopoietic malignancies. Best results are achieved when the underlying neoplasm has been turned into a stage of minimal disease by chemotherapy. Allo-HSCT in advanced solid tumors including pancreatic cancer have been of limited success, however studies of allo-HSCT in solid tumors in minimal disease situations have never been performed. The aim of this trial is to provide evidence for the clinical value of allo-HSCT in pancreatic cancer put into a minimal disease status by effective surgical resection and standard adjuvant chemotherapy.

**Methods/Design:**

The STEM PACE trial is a single center, phase II study to evaluate adjuvant allogeneic hematopoietic stem cell transplantation in pancreatic cancer after surgical resection. The study will evaluate as primary endpoint 2 year progression free survival and will generate first time state-of-the-art scientific clinical evidence if allo-HSCT is feasible and if it can provide long term disease control in patients with effectively resected pancreatic cancer. Screened eligible patients after surgical resection and standard adjuvant chemotherapy with HLA matched related stem cell donor can participate. Patients without a matched donor will be used as a historical control. Study patients will undergo standard conditioning for allo-HSCT followed by transplantation of allogeneic unmanipulated peripheral blood stem cells. The follow up of the patients will continue for 2 years. Secondary endpoints will be evaluated on 7 postintervention visits.

**Discussion:**

The principal question addressed in this trial is whether allo-HSCT can change the unfavourable natural course of this disease. The underlying hypothesis is that allo-HSCT has the capacity to provide long-term disease control to an extent otherwise not possible in pancreatic cancer, thereby substantially improving survival of affected patients.

**Trial registration:**

This trial has been registered: ISRCTN47877138

## Background

### Rationale

Pancreatic cancer is one of the major causes of cancer death globally, with a 5-year survival rate of less than 5% [1]. In the western world pancreatic cancer is the third most common cancer related cause of death. Even though substantial research was conducted in the last decades, to the current date, surgical resection is the only effective therapy that offers significant survival prolongation and potential cure of patients with pancreatic cancer. The outlook of patients who undergo surgical resection is better and specialized centers can achieve a resection rate of about 15% [2]. Although surgical resection cannot guarantee a cure, 5-year survival is improved to around 10% after resection [2]. Therefore an obvious need exists to improve the long-term survival in pancreatic cancer patients even in patients resected with curative intent. While it remains unclear if there is a survival benefit of adjuvant chemoradiotherapy with or without maintenance chemotherapy [3-6], there is a survival benefit for adjuvant chemotherapy [5,7-11].

Whereas overall survival of patients with ductal adenocarcinoma remains generally low, some patients with long-term survival and improved prognosis have been identified previously [2,12]. Prognosis of patients with pancreatic adenocarcinoma is defined and was later validated by tumor stages according to the AJCC Cancer Staging Manual [13]. In patients with resected pancreatic adenocarcinoma prognostic factors were recently defined in a cohort of patient undergoing a highly standardized surgical strategy [14]. In this study tumor size, nodal status and distant metastasis were confirmed as independent predictors of survival in patients who underwent resection. Additional independent negative prognostic parameters were identified in our patient cohort being age >70 years, preoperative presence of insulin-dependent diabetes mellitus, serum CA-19-9 levels above 400 U/ml, G3/4 tumor grading and a lymph node ratio (LNR) >0.2 [14]. Also, the revised R1 resection classification was an independent predictive marker [14]. G1 tumor grading was identified as positive risk factor [14] (Table 1).

**Table 1:** **Negative and positive predictors for overall survival in patients undergoing pancreatic resection according to Hartwig et al **[14]

**Prognostic factors for overall survival**	
**Negative predictors (weighted + 1)**	**Positive predictors (weighted − 1)**
Age >70 years	G1 tumor grading
Preoperative IDDM	Tis/T1/T2
CA-19-9 > 400 U/ml	R0 revised
G3/4 tumor grade	
LNR >0.2	
M1-status	
T4-status	

Using this prognostic risk score (“Hartwig Score”), the survival between prognostic groups can be further discriminated additionally to the AJCC staging system, identifying 4 risk groups with 5-year survival rates of 0%, 6.6%, 17.4% and 54.6% from the highest to the lowest risk group. This risk grouped analysis provided additional information especially for the largely heterogeneous group of AJCC stage II patients.

Altogether, this still indicates that there is a clear need to improve the long-term survival in pancreatic cancer patients in high risk groups (group 1–3) even in curatively resected patients asking for novel treatment strategies.

### Allogeneic hematopoietic stem cell transplantation in solid tumors

As the field of cancer immunology has grown, a deeper understanding of the immune system’s recognition of tumor cells and their antigens has translated into exciting new treatments for a variety of solid tumors including pancreatic cancer. The main function of the human immune system lies in the identification and elimination of pathogenic organisms such as bacteria and viruses but also of the body’s own defective cells, such as tumor cells. During cancer development, e.g. pancreatic cancer, the immune system apparently fails to identify and reject those tumor cells. To activate the partially “blind” immune system of cancer patients to fight against the tumor has been a goal in cancer research for decades. In this study we aim to exchange the unresponsive immune system with a new, tumor-sensitive immune system by allogeneic hematopoietic stem cell transplantation (allo-HSCT).

Transplantation of allogeneic hematopoietic stem cells from a human-leukocyte-antigen (HLA)-compatible donor is an established treatment for relapsed and high-risk hematological malignancies [15]. Allogeneic transplantation in hematological neoplasms can provide eradication of disease by a donor T-cell mediated immune graft-versus-tumor (GvT) effect, which also may be effective in solid tumors [16].

Allo-HSCT for a solid tumor was initially published for a patient with breast cancer [17]. To date, several series of allogeneic transplants in solid tumors have been published aiming at exploiting the GvT effect especially in breast and in renal cancer [18]. These concepts have been complemented with the development of nonmyeloablative (reduced intensity) allogeneic transplantation with or without the use of donor lymphocyte infusions (DLI) to avoid the high treatment related mortality and morbidity associated with the use of conventional myeloablative conditioning regimens [18]. Currently more than 1000 patients with refractory or other advanced solid tumors have undergone this treatment option [18]. Complete or partial responses have been achieved in patients with several types of solid tumors, including renal cell [19-23], breast [22-25], ovarian [23], colon [19,26,27] and also pancreatic cancers [28-31].

There are several lines of evidence that GvT effects are effective in solid tumors. It was shown that survival in patients with solid cancer was significantly improved in patients with chronic GVHD, suggesting GvT activity [23]. In a study in renal cancer, chronic GVHD and DLI were associated with improved survival [32]. Additionally DLI efficacy was observed after allo-HSCT for breast cancer [22-25]. Regression of renal cancer after allo-HSCT was correlated with the emergence of tumor antigen-reactive T cells [33], and the expansion of tumor-specific T cells could be demonstrated in patients who responded after GVHD onset following allo-HSCT for colon carcinoma [19,26,27].

### Allo-HSCT in pancreatic cancer

Although pancreatic cancer was originally thought to be poorly immunogenic, recent data have challenged this presumption. First, a high incidence of tumor-specific T lymphocytes is seen in bone marrows of patients with pancreatic cancer [34]. Second, Fukunaga et al. [35] analyzed 80 surgically resected pancreatic cancer tumors looking specifically for CD4^+^ T cells, CD8^+^ T cells, and dendritic cells within the tumor [35]. They reported that higher levels of CD4^+^ and CD8^+^ tumor-infiltrating lymphocytes in pancreatic cancers were associated with longer overall survival after surgical resection. The presence of both CD4^+^ and CD8^+^ tumor-infiltrating lymphocytes was an independent favorable prognostic factor in a multivariate analysis [35]. These data, together with results from early immunotherapy clinical trials, support the hypothesis that pancreatic cancer is treatable by an antitumor immune response. In this regard, Omuro et al. reported a marked regression of a large pancreatic tumor following allogeneic transplantation, which was attributed to GvT effects due to complete T-cell chimerism before tumor regression [31]. Recently, Abe et al. evaluated 5 patients with advanced pancreatic cancer who received allo-HSCT [28]. In these patients complete donor chimerism was obtained in 3 out of 5 patients. Antitumor effects considered to be GVT-mediated were observed in 2 of 5 patients.

### Limitations of allo-HSCT in solid tumors

However, in contrast to hematopoietic malignancies, complete and durable regressions of solid tumors have been observed only very rarely after allo-HSCT. This could be due to genuine biological differences between hematopoietic and epithelial tumor cells, such as expression of HLA-class-II or minor histocompatibility antigens [36], or the protective effect of tumor stroma regularly present in formations of solid tumor cells [36].

Apart from these biological considerations one obvious reason for the yet overall disappointing results of allo-HSCT for solid tumors could be the fact that almost all published studies have been performed in patients with advanced disease characterized by bulky/metastatic and actively proliferative tumor masses. It is well known from multiple studies in hematopoietic malignancies that the probability of effective GvT-mediated disease control after allo-HSCT is strongly correlated with tumor mass and proliferation at the time of transplant [36-38]. Thus, allo-HSCT may be much more effective if performed in a situation of temporarily controlled minimal residual disease. Notably, tumor mass and proliferation activity were found to be a strong survival predictor in studies on allo-HSCT for solid tumors [23,32].

### Rationale for a trial on allo-HSCT in pancreatic carcinoma in a MRD setting

Thus, the hypothesis underlying this trial is that the GvT activity conferred with allo-HSCT could result in complete eradication of pancreatic adenocarcinoma if applied in a setting of minimal residual disease achieved by radical resection of the primary tumor, thereby curing the disease. This is based on the following considerations:

•There is a large body of pre-clinical and clinical evidence that GvT can be effective in solid tumors including pancreatic cancer;

•In solid tumors, factors protecting tumor cells from GvT activity, such as an unfavorable effector-target cell ratios, immunosuppressive effects derived from large tumor masses, and a protective stromal environment, could be circumvented by applying allo-HSCT in an MRD setting;

•Broad and reproducible evidence from allotransplants in hematopoietic malignancies shows that allo-HSCT is indeed much more capable of providing durable disease control if performed in an MRD setting than in the presence of bulky and uncontrolled tumor masses.

### Objectives

The overall objective of this trial is to generate for the first time state-of-the-art scientific clinical evidence that allo-HSCT can provide long-term disease control in patients with radically resected pancreatic ductal adenocarcinoma and may have the potential to change the natural course of this otherwise fatal malignancy.

Specifically, the study aims at demonstrating that allo-HSCT is feasible in patients with radically resected pancreatic adenocarcinoma, and at exploring if the immunotherapeutic activity conferred with allo-HSCT can provide anti-tumor efficacy in the setting of minimal residual carcinoma persisting after surgical resection.

## Methods/Design

### Trial location

The trial will be performed at a single site located at the University Hospital Heidelberg, Germany. All patients will be operated at the Department of General, Visceral and Transplanation Surgery, and allo-HSCT will be performed at the Department of Internal Medicine V.

### Study design

This is a single-arm, open phase II trial comparing the study data to historical controls. Sponsor of the trial according to the GCP [39] guidelines and the German AMG regulations is the University Hospital Heidelberg.

The duration of intervention per patient will be 5 weeks. On protocol follow-up time per patient is 2 years after surgical resection.

Surgical resection and adjuvant chemotherapeutic treatment prior to study intervention (i.e. allo-HSCT) will follow accepted standards but is not part of the protocol.

### Trial population and eligibility criteria

Candidate patients have successfully undergone standard surgical treatment for pancreatic cancer followed by accomplishment of standard adjuvant chemotherapy. Patients, who have one or more full siblings, will be screened for eligibility at the Department of General, Visceral and Transplantation Surgery before discharge from hospitalization for tumor resection. Screening will be documented on the screening log, but screening results will be documented on CRF only if the patient is subsequently registered.

### Inclusion criteria

•Histologically proven diagnosis of pancreatic ductal adenocarcinoma having undergone radical resection (R1/R0 local resection) within the last 4–6 months at the University Hospital Heidelberg

•Hartwig score 1 or 2 (Hartwig et al. [14])

•Measurable tumor serum marker (i.e. CA 19–9) prior to resection

•Age at registration 18 to 65 years

•Karnofsky index > /= 70

•Hematopoietic cell transplantation comorbidity index (HCT-CI) score 0–1 (pancreatic carcinoma does not count against the score)

•HLA-identical (10/10 intermediate-resolution) related donor

•Written informed consent, signed and dated

### Exclusion criteria

•Hartwig score ≤ 0 (Hartwig et al. [14])

•HIV, HBV, HCV seropositivity

•Organ dysfunction

–Symptomatic coronary artery disease or ejection fraction <35%

–DLCO ≤60%, FEV1 < 65% of predicted FEV1 despite appropriate treatment or receiving supplementary continuous oxygen

–Liver function abnormalities: Patients with will be excluded if total serum bilirubin >1.5 X ULN, or AST/ALT >2.5XULN

–Chronic renal dysfunction defined by a creatinine clearance <50 ml/min.

•Fertile men and women unwilling to use contraceptive techniques during and for 12 months following treatment

•Females who are pregnant or breastfeeding

•Active other malignancies and/or a history of another malignancy treated by chemotherapy or radiotherapy within the last five years prior to inclusion

•Patients with systemic, uncontrolled infections

•Current alcohol or drug abuse

•Previously known contraindication and/or intolerance against study-related substances including medication for immunosuppression

•Inability to understand the scope of the study and intent of treatment. Dementia or altered mental status that would prohibit understanding informed consent

•Participation in another interventional clinical trial according to the German Pharmaceuticals Act (AMG) within 30 days prior to inclusion

### Gender consideration

The distribution of female and male patients is not relevant for the study as both gender are affected by pancreatic adenocarcinoma and treatment effects are not different in both groups.

### Recruitment and trial timeline

The duration of the trial for each subject is expected to be 24 months (one month study intervention and 23 months follow-up). The overall duration of the trial is expected to be approximately 45 months. Recruitment of subjects will start in April 2013. The actual overall duration or recruitment may vary and is estimated to be completed in September 2014.

### Sample size and statistical consideration

The trial will include 12 patients undergoing the study intervention. Patients who were registered on the basis of meeting the eligibility criteria but cannot proceed to allo-HSCT because of donor ineligibility, refusal, disease progression, comorbidity or interim ineligibility according to the inclusion/exclusion criteria will be replaced and will not be counted against the accrual target of 12 patients.

For safety reasons, patients will be enrolled in a 3 + 3 + 6 sequence: After registration of the first 3 patients, enrolment will be interrupted until the review of 100-day NRM and SAE reported for these 3 patients by the Data Monitoring and Safety Committee (DMSC) has confirmed safety thus justifying continuation of the trial. A similar procedure is repeated after the next 3 patients accrued, and before the remaining 6 patients can be enrolled. The 100-day NRM for this trial is anticipated to be 20% or less, calculated on the basis of all 12 patients to be included.

The trial is designed to explore the feasibility of the investigated therapy. If the results of this study indicate feasibility, it will serve as a basis for larger randomized protocols for proving efficacy. As no formal statistical hypotheses are defined, the analysis is performed by methods of descriptive data analysis, and therefore, no formal sample size calculation is performed.

### Investigational plan

#### Screening and pre-information

**Figure 1:**
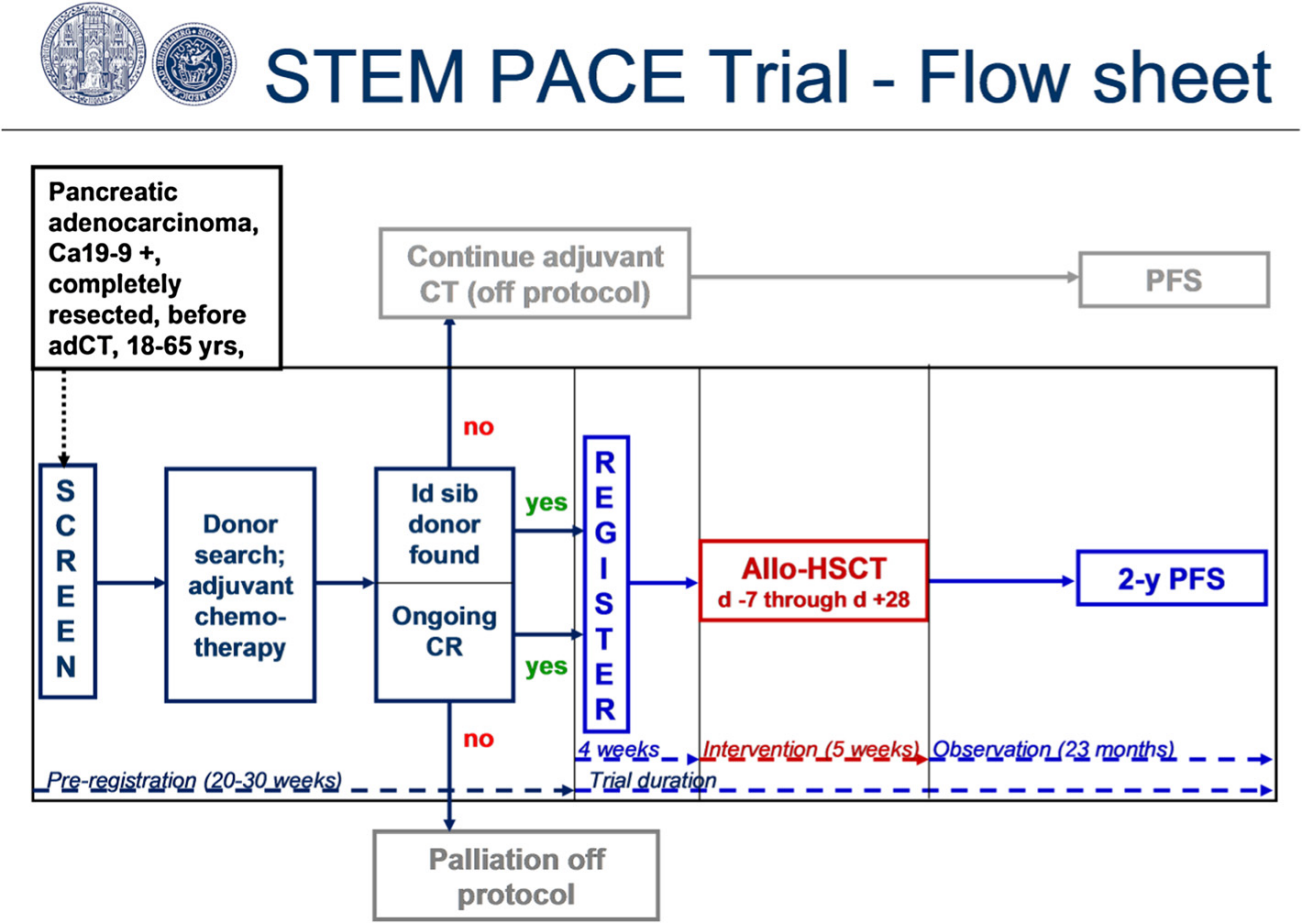
Simplified study investigational plan.

A simplified flow sheet of the trial can be found in Figure 1. Candidate patients will have successfully undergone standard surgical treatment for pancreatic cancer followed by accomplishment of standard adjuvant chemotherapy, and who have one or more full siblings, will be screened for eligibility. During the screening procedure, the patient will be concisely informed about the possibility of participating in the trial and its purpose, principle, risks, and chances. He/she will be explained that a prerequisite for participation is the need for donor search, and that registration with the trial is possible only if a matched related donor is found. The patient will be informed that probability of meeting this criterion is 25% per sibling.

Patients willing to initiate donor search have to confirm their consent to this in written form.

### Donor search

Sibling donor search will be initiated immediately after signing the informed consent form by the patient. Siblings will be contacted by standard procedures, provided with written information briefly describing the trial and the need to obtain a blood sample from them, which is to be submitted along with the written and undersigned consent form for HLA-typing to the Coordination office. HLA-A, B, C, DRB1, and DQB1 typing is to be performed using DNA-based methods. To be eligible for inclusion, patients and related donors have to be HLA-identical (10/10 intermediate-resolution). A matched related donor may not have any a priori contraindications and a donor written informed consent will be obtained by an independent investigator to avoid conflicts of interest.

### Patient registration and informed consent

Once the patient has completed 12–20 weeks of adjuvant chemotherapy without disease progression and has a matched related stem cell donor available, he/she will undergo routine follow-up assessment. Prior to registration, eligible patients will be provided with information in comprehensible terms about radically resected pancreatic ductal adenocarcinoma and the current status of knowledge about treatment of this disease and on the aims of the study, and about the possibility to participate in the present trial. It will be explained to the patient that once she/he has started conditioning chemotherapy for allo-HSCT, any refusal of subsequent parts of the study intervention (such as transplantation or use of immunosuppressive drugs) will be associated with a very high risk of potentially life-threatening complications and is strongly discouraged.

In a second independent oral explanation at the Department of Hematology, Oncology and Rheumatology, interested patients will have the chance to sign the informed consent.

Eligible patients will then be registered for the trial.

After registration, donors will be invited for an explanatory interview to ensure that the donor is fully aware that he/she is free to decide whether to participate or not, that he/she can cancel his/her decision to participate at any time and that there will be no disadvantages for her/him in case of refusal. However, it will be explained to the donor that once she/he has successfully completed the routine work-up qualifying for activation of the patient conditioning process for allo-HSCT (“final donor clearance”), refusal of stem cell donation will be associated with a very high risk of potentially life-threatening complications for the patient. Donors will subsequently sign the informed consent in case of study participation.

### Randomization and blinding

No randomization and blinding will be part of this clinical trial.

### Study intervention

After registration, patients will undergo routine work-up for allo-HSCT (not to be reported on CRF) and should proceed to admission for transplantation within four weeks from enrolment. This interval may be prolonged because of logistic (e.g. donor availability) or medical reasons (e.g. treatment of infectious foci). Duration of study intervention is defined as the interval from the day before start of conditioning (d −7) and d +28 after allo-HSCT.

### Conditioning

Patients will be subjected to conditioning for allo-HSCT according to the following conditioning regimen:

### Fludarabine/Cyclophosphamide

day −6 through day −2: Fludarabine 30 mg/m^2^/d (=150 mg/m^2^ total dose)

day −3 and day −2: Cyclophosphamide 60 mg/m^2^/d (=120 mg/kg total dose)

### Stem cell product and stem cell transplantation

Only mobilized hematopoietic stem cells obtained from peripheral blood by hemapheresis according to § 13 AMG and the German Transfusion Law will be used for the purposes of this trial. The use of hematopoietic stem cells harvested from bone marrow according to § 20 b AMG and § 8 Transplantation Law is not allowed in this trial.

Stem cell harvesting and production of the allograft will be exclusively provided by the IKTZ Heidelberg (partly using facilities of the Department of Hematology, Oncology and Rheumatology) by standard procedures.

Fresh unmanipulated G-CSF-mobilized peripheral blood stem cells (PBSC) obtained from the matched related donor by standard leukapheresis are used as stem cell source. A PBSC dose of more than 4 × 10^6^ CD34 + cells/kg body weight is recommended. If this target cannot be reached by two leukapheresis procedures on consecutive days, the transplanted cell dose will be limited to the amount obtained at that time.

Transplantation is performed on day 0 according to approved standard operating procedures.

### GVHD prophylaxis and immune modulation

GVHD prophylaxis should be carried out with cyclosporine A (CSA) and mycophenolate mofetil (CSA/MMF). MMF treatment will then continue through day +30 and will be discontinued without a taper if GVHD is absent. CSA will be given from day −1 onwards.

If active GVHD is absent, CSA taper might begin from day +60 onwards (25% of original dose every 14d). The recommended schedules for administration of immunosuppressive drugs may be altered according to GVHD, chimerism and MRD kinetics.

Donor lymphocyte infusion (DLI) is not formal part of the study intervention but may be considered for patients with incomplete chimerism and/or stable or increasing MRD levels in the absence of GVHD not earlier than 4 weeks after complete withdrawal of systemic immunosuppression. The chimerism is tested during the indicated follow up visits (Additional file 1: Table S1) and analyzed by short-tandem-repeat PCR [40]. The recommended T cell starting dose is 5x10^6^/kg recipient weight. DLI might be repeated every 8–12 weeks with 3-fold increases of T cell number as long as MRD kinetics is unaltered and GVHD is absent. T cells for DLI should be obtained from the PBSC donor without prior G-CSF mobilization by leukapheresis according to IMPD after separate written informed consent.

Supportive measures during and after allo-HSCT will be administered according to approved standard operating procedures and are not part of the study intervention.

### Endpoints

#### Primary endpoint

Primary endpoint is to study if RIC allo-HSCT can provide long-term freedom from disease recurrence with radically resected pancreatic adenocarcinoma (measured as 2-year progression-free survival from registration, PFS). Events relevant for PFS are defined as clinical relapse, or death from any cause.

#### Secondary endpoints

Secondary efficacy endpoints are:

•2-year OS from registration;

•2-year PFS and OS after surgical resection;

•Minimal residual disease kinetics at day −28 (S), day 0 (R), day +28 day +56,, day +100, day +180, day +360, day + 540 and 720 days before/after allo-HSCT (MRD; measured by tumor serum marker levels) and their correlation with immune events (e.g. immunosuppression tapering, GVHD); Minimal residual disease will be measured by the tumor marker CA 19–9. The blood sampling will be performed routinely and the analysis will be done in the central lab.

•Impact of important explanatory variables (such as age, gender, comorbidity, revised R1 resection classification score, pre-transplant tumor marker levels, and other) on PFS and OS.

Secondary feasibility endpoints are:

•Non-relapse mortality (NRM) at 3 and 24 months after allo-HSCT. Events relevant for NRM are defined as death from any cause in the absence of first disease recurrence after surgical resection;

•Prevalence of chronic graft-versus-host-disease at 6, 12 and 24 months from allo-SCT (cGVHD; measured by NIH consensus project forms);

•Quality of life at day −28 (S) , day +28, day 56, day +100, day +180, day +360, day +720 (end of study) before/after allo-HSCT (QOL; measured by EORTC QLQ C30 and HDC29);

•Impact of important explanatory variables (such as age, gender, comorbidity, revised R1 resection classification score, pre-transplant tumor marker levels, and other) on NRM.

### Response evaluation

Clinical response will be determined by routine tumor follow-up according to the RECIST criteria. Tumor follow-up will be performed at screening, Month 3, 6, 12, 18, and 24 following routine clinical practice.

Karnofsky index and tumor marker CA19-9 will be determined at screening, at inclusion (Day 0), on Day 28, and at each of the follow-up visits

### Risks

To archive the benefit of a potential cure, the risk of the allo-HSCT needs to be taken into account. With allogeneic HSCT the risk remains, that the GVHD becomes so severe, that it cannot be controlled by immunosuppression. Additionally, there remains a risk of severe infections. Both side effects and complications can reduce the quality of life and can be lethal. Also pancreatic cancer may relapse even under allo-HSCT. The treatment (allo-HSCT) related mortality remains under current regimens around 10-20% within the first 24 months.

### Study visits

There will be 2 pre-intervention study visits, (1) screening, (2) registration. After the intervention 7 follow up visits are planned on d28, d56, d100, d180, d360, d540 and d720. The visits will asses eligibility criteria, informed consent, HLA typing, performance scores, blood sampling, abdominal CT scans, relapse evaluation, acute and chronic GVHD evaluation, quality of life, adverse event and serious adverse event recording. For details see Additional file 1: Table S1.

After the end of the study or if the study finished prematurely, the treatment of the patient is left to the discretion of the responsible (treating) physician and is conducted according to medical standards. Patients will be continously monitored also after the end of the study.

### Data management

All protocol-required information collected during the trial must be entered by the investigator, or a designated representative, in the CRF. The investigator, or the designated representative, should complete the CRF pages as soon as possible after information is collected. Any outstanding entries must be completed immediately after the final examination. An explanation should be given for all missing data. Completed CRF must be reviewed and signed by the investigator or by a designated sub-investigator. CRFs are sent to the Institute of Medical Biometry and Informatics Heidelberg (IMBI) for data entry. Copies remain at the trial site.

In order to ensure that the database reproduces the CRFs correctly, the IMBI accomplishes double data entry. The completeness, validity and plausibility of data are examined by validating programs, which thereby generate queries. The investigator or the designated representatives are obliged to clarify or explain the queries. At the end of the trial, the principle investigator will retain the originals of all CRFs.

The data will be managed and analyzed in accordance with the appropriate SOPs valid in the IMBI.

### Monitoring

Monitoring will be done by personal visits from a clinical monitor according to SOPs of the KKS (Coordination Centre for Clinical Trials Heidelberg). The monitor will review the entries into the CRFs on the basis of source documents. Frequency and details of monitoring will be defined in the monitoring manual. The investigator must allow the monitor to verify all essential documents and must provide support at all times to the monitor.

By frequent communications (letters, telephone, fax), the site monitor will ensure that the trial is conducted according to the protocol and regulatory requirements.

### Safety evaluation, analysis and reporting

An *adverse event* (AE) is any unwanted medical occurrence in a patient or clinical investigation subject administered a pharmaceutical or medical product and which does not necessarily have a causal relationship with this treatment.

A serious adverse event (SAE) is any adverse event occurring at any time during the period of observation, that results in death, is life-threatening, causes inpatient hospitalization of prolongation of hospitalization, results in persistent or significant disability or incapacity, leads to a congenital anomaly or is otherwise medical relevant.

In order to investigate safety and tolerability of the treatment, following parameters will be collected in the course of the trial: adverse events (AEs), laboratory parameters (CBC, clinical chemistry), Clotting factors (INR and Quick), haematology, AST, ALT, electrolytes, thyroid value, ferritine and evaluations on acute and chronic GVHD. Furthermore, the continuing monitoring of safety of the enrolled subjects will be assured by collecting and processing of serious adverse events (SAEs). So as to obtain an independent expert opinion a Data Monitoring and Safety Committee (DSMC) will be appointed.

All AEs will be carefully documented on the appropriate pages in the CRF. Undesirable signs, symptoms or medical conditions/diseases present before starting study treatment are only to be documented if they worsen after starting study treatment. The start date for AEs that were present at the study start and that worsen during the study should be reported as the date the events worsened, not the date the events began pre-study.

Only SAEs should be reported and – on separate forms – any symptoms or signs of acute or chronic GVHD even if not fulfilling the criteria of SAE. SAEs will be documented up to the last trial visit, i.e. up to 24 months after trial inclusion.

However, certain adverse events are a mandatory consequence of the conditioning regimen administered prior to allo-HSCT. Therefore any changes in blood counts and differential between days −7 and +28 will be monitored and captured by CRF but should not be recorded additionally as AE.

If an SAE occurs, the investigator completes the SAE report form. The form has to be sent (by fax) to the Trial Pharmacovigilance Office within 24 hours.

The Trial Pharmacovigilance Office notifies the Paul-Ehrlich-Institute (reports to the Federal Ministry of Health), the Ethics Committee and the PI of any SUSAR according to the prevailing regulations.

### Data Monitoring and Safety Committee (DMSC)

An independent Data Monitoring and Safety Committee (DMSC) has been set up in accordance with the European “Guideline on Data Monitoring Committees” (EMEA/CHMP/EWP/5872/03 Corr).

The DMSC consists of three independent members, who will not be involved in the trial. One is a senior biostatistician, one is an expert in surgical RCTs and one is an expert in medical RCTs. All have served already on DMSCs. At pre-specified time-points, namely after enrollment and day +100, assessment of the first and second patient cohorts of 3 patients each, respectively, the DMSC will evaluate 100-day NRM and SAE status. Based on the results of these two safety evaluations, the DMSC will recommend continuation, interruption, amendment or termination of the trial. In addition, the DMSC may recommend interruption of the trial at any time if safety concerns arise upon SAE evaluation or due to other reasons.

### Statistical analysis

The primary and secondary outcomes will be analyzed applying methods of descriptive data analysis.

For the primary endpoint, 2-year progression-free survival after surgical resection, the Kaplan-Meier estimate and the corresponding two-sided 90% Greenwood confidence interval based on a log(−log)-transformation [31] are calculated. The primary analysis is performed based on the full analysis set; to assess the impact of major protocol deviations, the analyses of the primary endpoint described above will also be performed for the per protocol set.

Analysis of the secondary endpoint, overall survival, will be performed analogously to the primary endpoint. Furthermore, all documented variables will be analyzed descriptively by tabulation of the measures of the empirical distributions. According to the scale level of the variables, mean, standard deviations, median, 1^st^ and 3^rd^ quartile, as well as minimum and maximum or absolute and relative frequencies, respectively, will be reported. Additionally, for variables with longitudinal measurements the time course of individual patients will be depicted. Descriptive p-values of the corresponding statistical tests comparing baseline and follow-up measurements and associated 95% confidence intervals will be given.

Safety analysis will be based on the data set of all patients who were treated within the trial. The safety analysis includes calculation of frequencies and rates of reported adverse and serious adverse events.

An important part of the analysis comprises the comparison of the results of this trial with historical data available for the patient population under consideration. At the timepoint of the analysis, a systematic review will be performed providing the up-to-date evidence on efficacy and safety of alternative therapies.

All analyses will be done using SAS version 9.1 or higher.

### Funding

Heidelberg Surgery Foundation, University of Heidelberg, Im Neuenheimer Feld 110, D-69120 Heidelberg, Tel: (49) 06221/ 56 4875, Fax: (49) 06221/56 4877, stiftung.chirurgie@med.uni-heidelberg.de, http://www.stiftung-chirurgie.com.

### Ethical and legal consideration

#### Approval

In accordance with the Declaration of Helsinki, the German Drug Law (Arzneimittelgesetz [AMG]), the German Good Clinical Practice ordinance (GCP-V), and the Note for Guidance on Good Clinical Practice (GCP) [39], the study was presented to the independent Medical Ethics Committee of the University of Heidelberg (EC) and the Paul-Ehrlich-Institute (PEI), as competent authority. The approval of the EC and the authorization of the PEI will be obtained prior to any study-related procedures.

Any substantial change in the clinical study protocol and any change in the informed consent form (§ 10 and § 11 GCP-V) will be presented to the EC and any substantial change in the clinical study protocol to the PEI. Substantial amendments have to be approved by them before implementation (except to eliminate immediate hazards).

The EC and the PEI will be informed about the end of the study (§ 13 GCP-Verordnung).

### Patient informed consent

According to AMG, GCP-V, and the Note for Guidance on GCP [39], written informed consent must be obtained from patients prior to participation in the study.

Patients and donors will voluntarily and separately confirm their willingness to participate in the study, after having been informed by separate physicians in writing and verbally of all aspects of the study that are relevant to their decision to participate. They will be informed about requirements concerning data protection and have to agree to the direct access to their individual data.

Patients will be informed that they are free to withdraw from the study at any time at their own discretion without necessarily giving reasons.

### Good clinical practice and other legal basis

The study will be carried out in conformity with the principals of the Declaration of Helsinki and the Guidelines for Good Clinical Practice in their current revisions. The trial will be carried out in keeping with national and international legal and regulatory requirements.

### Registration

http://www.controlled-trials.com/ISRCTN47877138

## Discussion

The STEM PACE study aims to generate state-of-the-art scientific clinical evidence that reduced intensity allo-HSCT is feasible in patients with effectively resected pancreatic adenocarcinoma and, further, that it can provide long-term disease control. This approach is aimed to elucidate if allo-HSCT under conditions with minimal residual disease may have the potential to change the natural course of this, even with modern chemotherapy regimens, otherwise mostly fatal disease. Due to the severe side effects of allo-HSCT, including high levels of treatment related mortality in the patients, this therapy option was up to now mainly explored in patients with refractory and far advanced solid tumors. Even though some of the results from previous studies were promising with objective tumor response in refractory disease settings, treatment of solid tumors with allo-HSCT remains in a niche.

The STEM PACE study will evaluate the role of allo-HSCT using a novel approach in solid tumors. To circumvent the treatment related mortality of standard myeloablative regimens we will apply a non-myeloablative reduced-intensity conditioning regimen. This use of non-myeloablative conditioning does therefore not rely on chemotherapy related cytoreduction administered before transplant but rather on the immune effect of the grafted stem cells. Non-myeloablative conditioning before allogenic transplantation can archive full myeloid and lymphoid chimarism leading to anti-tumor activity with a substantial reduction of treatment related mortality [18,41,42]. Using this technique, the study will rely on the immune graft-versus-tumor effect of the allo-HSCT. This effect is well described in hematological diseases in which patients with graft-versus-host disease have a lower probability of relapse than patients without. Additional donor lymphocyte infusions were able to successfully induce remission in relapsed patients without additional cytotoxic therapy after allo-HSCT [36]. Although it is not well investigated to which extent the pancreas is a target organ for chronic GVHD or potential GvT, preliminary evidence suggests the pancreatic gland might be affected by acute or chronic GVHD [43,44]. Moreover, it is well acknowledged that the biologically closely related salivary glands are a prime target organ for chronic GVHD prone to complete destruction by GVHD-mediated inflammation [45].

However, it needs to be taken into account that in previous studies a high proportion of patients who underwent allo-HSCT in solid tumors progressed rapidly due to the high initial tumor burden. As described above, effective GvT-mediated disease control after allo-HSCT correlates with tumor mass and proliferation at the time of transplant [36-38]. This remains a special problem in pancreatic cancer as a fast progressing tumor. To overcome this limitation we will only include patients who have undergone surgery and adjuvant chemotherapy, leading to a minimal residual disease setting. Under these conditions we hope to achieve a full chimarism and GVT before relapse of the disease. This might give the opportunity to eliminate remaining tumor cells, which will otherwise most likely lead to recurrence of the tumor. Transplantation after surgery with subsequent chemotherapy will additionally allow the chance to select patients with at least stable disease, therefore identifying a more suitable patient population who might profit from this treatment. This is of special importance since it was published before that out of 116 patients with different solid tumors response to allo-HSCT was strongly correlated to stable disease, while only 1 out of 52 patients with progressive disease showed a clinical response [23].

The strong stromal reaction of pancreatic cancer might pose an additional problem for allo-HSCT in advanced pancreatic cancer, which will be bypassed by surgical tumor removal. This should be taken into account, since in leukemias the stromal microenvironment acts in a protective manner to malignant cells by different mechanisms [46-48]. By interaction with the microenvironment, hematopoietic tumor cells persisting at extramedullary sites seem to less sensitive to GvT effects [36,49].

The role of allo-HSCT in pancreatic cancer was previously studied [29-31], however, all previous studies were only done in relatively small cohorts. This phase II study will provide the opportunity to establish a clearer picture on the role of allo-HSCT in pancreatic cancer. As only fully HLA-matched directly related sibling donors will be included into this trial it will be possible to compare the outcome of this study with a non-randomized control group without undergoing a positive selection bias. The selection and recording of the primary and secondary endpoints will allow further phase II or III studies in the future in case of promising results. With the focus on a single center the standardized surgical procedures as well as the pathological evaluation will be comparable at this phase of clinical trials. The pathologic postoperative evaluation is of importance in pancreatic cancer, as the tumor infiltration of the resection margin, the R status, is especially difficult to distinguish and often wrongly assessed as a R0 resection [50].

To discuss the ethical implications of treating surgically resected patients after chemotherapy with an allo-HSCT, the overall survival of pancreatic cancer patients needs to be taken into account. Even patients who have undergone the best current treatment with complete surgical resection and adjuvant chemotherapy only achieve a median survival of about 24 months [5,8,9]. This unfavorable outcome asks for further alternative treatment options [51]. On the other hand, using allo-HSCT as an additional treatment is accompanied with a risk of severe infections. Further, the risk remains, that the GVHD becomes so severe, that it cannot be controlled by immunosuppression. Both side effects and complications can reduce the quality of life and can be lethal. Treatment related mortality of allo-HSCT remains under current regimens around 10-20% within the first 24 months. Last but not least pancreatic cancer may relapse even under allo-HSCT. To take these treatment related risks into account we exclude patients with a high 5-year overall survival probability and pre-select patients with an average 5 year survival of below 20%. However this will need to be openly discussed with the patients additional to the patient information forms. In summary, allo-HSCT however risky, may provide potentially a chance to permanently cure or suppress pancreatic cancer. While 50% of all patients using the current available treatment will die within 2 years, allo-HSCT will lead to about 10-20% treatment related lethality in the patients and about the same amount of patients will have a reduced quality of life due to treatment related side effects (GvHD). In summary, we hope this treatment may offer a chance to eradicate persisting tumor cells in this otherwise lethal disease.

## Competing interest

The authors declare that they have no competing interests.

## Authors’ contributions

FHSW, TS, ML, PB, MK, ADH, PD and MWB designed the study. FHSW, TS, ML and PD wrote the study protocol and drafted the manuscript. MK provided the biostatistical study design. All authors read and approved the final manuscript.

## Pre-publication history

The pre-publication history for this paper can be accessed here:

http://www.biomedcentral.com/1471-2407/14/168/prepub

## Supplementary Material

Additional file 1: Table S1 Trial procedures and assessment overview. Click here for file
