# Supplementary material for: Stem cell Transplantation for Eradication of Minimal PAncreatic Cancer persisting after surgical Excision (STEM PACE Trial, ISRCTN47877138): study protocol for a phase II study
Source: BMC Cancer. 2014 Mar 10;14:168. doi: 10.1186/1471-2407-14-168 (PMC4008264; doi:10.1186/1471-2407-14-168)
Supplement: Additional file 1: Table S1 — Trial procedures and assessment overview. [file 1471-2407-14-168-S1.docx]

**TABLE S1**: Trial procedures and assessment overview

| Visit # | S | R | I | | FU 28 | FU 56 | FU 100 | FU 180 | FU 360 | FU 540 | E | | Routine follow-up  **(off study)** |
| --- | --- | --- | --- | --- | --- | --- | --- | --- | --- | --- | --- | --- | --- |
| Purpose | Screening | Registration | Study Intervention | | Safety/ efficacy assessment | | | | | | End of study | |  |
| Procedure | Start donor search | Register | Allo-HSCT | | Observe | | | | | | Observe | | Observe |
| Time | Before discharge after surgery | d -28 | d -7 through d +28 | | d +28 | d +56 | d +100 | Mo +6 | Mo +12 | Mo +18 | Mo +24 | |  |
| Informed Consent | Patient: Trial / HLA-Pre-Info  Siblings dito | Patient & Donor: Trial and donation |  | |  |  |  |  |  |  |  | |  |
| Medical History |  | X |  | |  |  |  |  |  |  |  | |  |
| Performance score (WHO Karnofsky) |  | X |  | | X | X | X | X | X | X | X | |  |
| HCT-CI score |  | X |  | |  |  |  |  |  |  |  | |  |
| CBC  (+ differential if ANC >1/nl) |  | X | -7, 0, +7, +10, +14, +17, +20, +24 | | X | X | X | X | X | X | X | |  |
| Chemistry |  | X (LDH, GPT, bilirubin, lipase, amylase, creatinine, glucose, CRP) | -7, 0, +7, +10, +14, +17, +20, +24 | | X | X | X | X | X | X | X | |  |
| Chimerism (PB) |  | X |  | | X | X | X | X | X | X | X | |  |
| Virology | X (CMV, HBV, HCV, HIV) | X (CMV, HBV, HCV, HIV) |  | |  |  |  |  |  |  |  | |  |
| Lung function test |  | X (if no pre-operative available) |  | |  |  |  |  |  |  |  | |  |
| Pregnancy test |  | X (women only) |  | |  |  |  |  |  |  |  | |  |
| Clotting factors (INR and Quick), haematology, AST, ALT, electrolytes, tyroidv value, ferritine | X |  | X | | X |  | X | X | X | X | X | |  |
| Blood sampling (for immune monitoring) |  | X | X | | X | X | X | X | X | X | X | |  |
| HLA typing | X |  |  | |  |  |  |  |  |  |  | |  |
| Abdominal  CT scan |  | X |  | | X |  | X | X | X | X | X | |  |
| Relapse |  |  |  | | X |  | X | X | X | X | X | |  |
| Tumor marker (e.g. Ca19-9) | X | X | -7, 0, +7, +14, +24 | | X | X | X | X | X | X | X | |  |
| Acute GVHD |  |  |  | Continuous documentation | | | |  |  |  |  | |  |
| Chronic GVHD |  |  |  | |  |  | Continuous documentation | | | | | |  |
| Immune modulation^1^ |  |  |  | Continuous documentation | | | | | | | | |  |
| Adverse events I-IV^2^ |  |  | X | | X |  |  |  |  |  | | |  |
| Serious AE^2^ |  |  | X | | X | X | X | X | If not related to relapse | | | |  |
| Quality of life  (EORTC) |  | X |  | | X | X | X | X | X | X | | X |  |

^1^ Dose modifications of systemic immunosuppression and donor lymphocyte infusions according to tumor marker and chimerism kinetics and GVHD.

^2^ Any changes in blood counts and differential between days -7 and +28 should not be recorded as AE. All other laboratory values out of normal range should be documented as AE only if clinical relevance is assumed by the investigator
